# Effects of glucose, insulin, and insulin resistance on cerebral ^18^F-FDG distribution in cognitively normal older subjects

**DOI:** 10.1371/journal.pone.0181400

**Published:** 2017-07-17

**Authors:** Kenji Ishibashi, Airin Onishi, Yoshinori Fujiwara, Kiichi Ishiwata, Kenji Ishii

**Affiliations:** 1 Research Team for Neuroimaging, Tokyo Metropolitan Institute of Gerontology, Tokyo, Japan; 2 Research Team for Social Participation and Community Health, Tokyo Metropolitan Institute of Gerontology, Tokyo, Japan; 3 Institute of Cyclotron and Drug Discovery Research, Southern Tohoku Research Institute for Neuroscience, Koriyama, Japan; 4 Department of Biofunctional Imaging, Fukushima Medical University, Fukushima, Japan; Nathan S Kline Institute, UNITED STATES

## Abstract

**Background:**

Increasing plasma glucose levels and insulin resistance can alter the distribution pattern of fluorine-18-labeled fluorodeoxyglucose (^18^F-FDG) in the brain and relatively reduce ^18^F-FDG uptake in Alzheimer's disease (AD)-related hypometabolic regions, leading to the appearance of an AD-like pattern. However, its relationship with plasma insulin levels is unclear. We aimed to compare the effects of plasma glucose levels, plasma insulin levels and insulin resistance on the appearance of the AD-like pattern in ^18^F-FDG images.

**Methods:**

Fifty-nine cognitively normal older subjects (age = 75.7 ± 6.4 years) underwent ^18^F-FDG positron emission tomography along with measurement of plasma glucose and insulin levels. As an index of insulin resistance, the Homeostasis model assessment of Insulin Resistance (HOMA-IR) was calculated.

**Results:**

Plasma glucose levels, plasma insulin levels, and HOMA-IR were 102.2 ± 8.1 mg/dL, 4.1 ± 1.9 μU/mL, and 1.0 ± 0.5, respectively. Whole-brain voxelwise analysis showed a negative correlation of ^18^F-FDG uptake with plasma glucose levels in the precuneus and lateral parietotemporal regions (cluster-corrected *p* < 0.05), and no correlation with plasma insulin levels or HOMA-IR. In the significant cluster, ^18^F-FDG uptake decreased by approximately 4–5% when plasma glucose levels increased by 20 mg/dL. In the precuneus region, volume-of-interest analysis confirmed a negative correlation of ^18^F-FDG uptake with plasma glucose levels (*r* = -0.376, *p* = 0.002), and no correlation with plasma insulin levels (*r* = 0.156, *p* = 0.12) or HOMA-IR (*r* = 0.096, *p* = 0.24).

**Conclusion:**

This study suggests that, of the three parameters, plasma glucose levels have the greatest effect on the appearance of the AD-like pattern in ^18^F-FDG images.

## Introduction

Increase of plasma glucose levels alters the distribution pattern of fluorine-18-labeled fluorodeoxyglucose (^18^F-FDG) in the brain. This phenomenon was first described by Kawasaki and colleagues in a positron emission tomography (PET) study with 19 cognitively normal elderly subjects, where glucose loading decreased ^18^F-FDG uptake especially in the precuneus, posterior cingulate, and lateral parietotemporal regions [1]. This relationship between increasing plasma glucose levels and decreasing ^18^F-FDG uptake in specific regions has been confirmed by Burns and colleagues, who showed that, in a cross-sectional PET study with 124 cognitively normal elderly subjects, plasma glucose levels were negatively correlated with ^18^F-FDG uptake in the precuneus and lateral parietal regions [2]. Interestingly, the precuneus, posterior cingulate, and lateral parietotemporal regions are associated with the representative hypometabolic areas preferentially observed in Alzheimer's disease (AD), suggesting that increasing plasma glucose levels induces the appearance of an AD-like pattern in ^18^F-FDG images. We have, thus far, investigated the effects of plasma glucose levels on the appearance of AD-like patterns. In the case of a 70-year-old man who had neither Aβ deposition nor ApoE ε4 genotype, the AD-like pattern appeared at plasma glucose levels of 162 mg/dL but disappeared at 106 mg/dL [3]. The AD-like pattern in ^18^F-FDG images can appear in cognitively normal older subjects with plasma glucose levels of 100 to 110 mg/dL [4], and also in young healthy subjects who received glucose loading [5, 6]. Additionally, increasing and decreasing plasma glucose levels induce the AD-like pattern reversibly to appear and disappear, respectively, in cognitively normal subjects with diabetes [7].

Higher insulin resistance is also able to alter the distribution pattern of ^18^F-FDG in the brain, leading to the appearance of the AD-like pattern in ^18^F-FDG images. Baker and colleagues conducted ^18^F-FDG PET scanning on cognitively normal subjects with prediabetes and early diabetes, and showed negative correlations between insulin resistance and ^18^F-FDG uptake in the precuneus, posterior cingulate, and lateral parietotemporal regions, which are the same as the AD-related hypometabolic regions [8]. Similar associations between higher insulin resistance and lower ^18^F-FDG uptake in specific areas, including AD-vulnerable regions, have been recently shown in patients with AD [9] and in middle-aged subjects at risk for AD [10].

Since an index of insulin resistance, Homeostasis Model Assessment of Insulin Resistance (HOMA-IR), is calculated using plasma glucose and insulin levels [11], the magnitude of insulin resistance has a close relationship with both plasma glucose and insulin levels. Although the AD-like pattern in ^18^F-FDG images can be associated with increasing plasma glucose levels and higher insulin resistance, the relationship between the AD-like pattern and plasma insulin levels is still unclear. The goal of this study was to compare the effects of plasma glucose levels, plasma insulin levels and insulin resistance on the cerebral ^18^F-FDG distribution, and to determine, of the three parameters, which one has the greatest effect on the appearance of the AD-like pattern. For this purpose, we performed ^18^F-FDG PET scanning on cognitively normal subjects as well as measuring both plasma glucose and insulin levels.

## Materials and methods

### Research participants

This prospective study was conducted in accordance with the Helsinki Protocol, and approved by the Ethics Committee of Tokyo Metropolitan Institute of Gerontology. Written informed consent was obtained from all 59 participants. Ten subjects were men and 49 were women, and the age range was 64 to 88 years. All subjects were recruited from ongoing longitudinal studies of cognition and aging at the institute, and completed an interview, physical and neurological examinations, a screening test for dementia, and magnetic resonance imaging (MRI) scanning. In an interview, all subjects were confirmed to have a conviction that they were cognitively normal and they were living an independent life without any assistance [12]. Subjects were excluded if their Mini-Mental State Examination (MMSE) score was less than 27, if their body mass index was less than 18.5 or more than 25.0, or if they had a history of diabetes. In physical and neurological examinations and a routine mental health interview performed by a neurologist, those with a neurological condition, a mental health condition, or any other uncontrolled health condition were also excluded. No subjects showed significant brain atrophy or lesions in the MRI findings. Finally, all subjects were deemed cognitively normal and healthy.

### Glucose and insulin levels

After more than 5 h of fasting, each participant underwent ^18^F-FDG PET scanning. Plasma glucose levels were measured twice, immediately prior to the intravenous ^18^F-FDG injection and 30 min after the injection, by using a medical device (Caresist; Horiba, Kyoto, Japan), and the two values were averaged. The measurement system for plasma glucose levels was based on the enzyme electrode method, which integrates hydrogen peroxide electrodes with a glucose oxidase immobilized membrane. Plasma insulin levels were measured once immediately prior to the ^18^F-FDG injection using a chemiluminescent immunoassay (BML, Tokyo, Japan). HOMA-IR was calculated as an index of insulin resistance by the following formula: HOMA-IR = [(fasting glucose level (mg/dL) × fasting insulin level (μU/mL)) / 405].

### PET scanning and image processing

The radioligand, ^18^F-FDG, was synthesized using a PET synthesizer (F300; Sumitomo Heavy Industries, Tokyo, Japan). The radiochemical purity of ^18^F-FDG was greater than 95%. The PET scanning was performed at the institute using the Discovery PET/CT 710 scanner (GE Healthcare, Milwaukee, WI) in the three-dimensional mode. The in-plane and axial resolutions of the full width at half maximum (FWHM) were 4.52 mm and 4.83 mm, respectively. CT-transmission data were acquired for measured attenuation correction. Emission data were acquired for 10 min starting at 40 min after an intravenous bolus injection of approximately 150 MBq (4 mCi) of ^18^F-FDG. Forty-seven-slice images with 2 × 2 × 3.27 mm^3^ voxel size and 128 × 128 matrix size were then obtained. Data were reconstructed after correction for decay, attenuation, and scatter.

**Fig 1:**
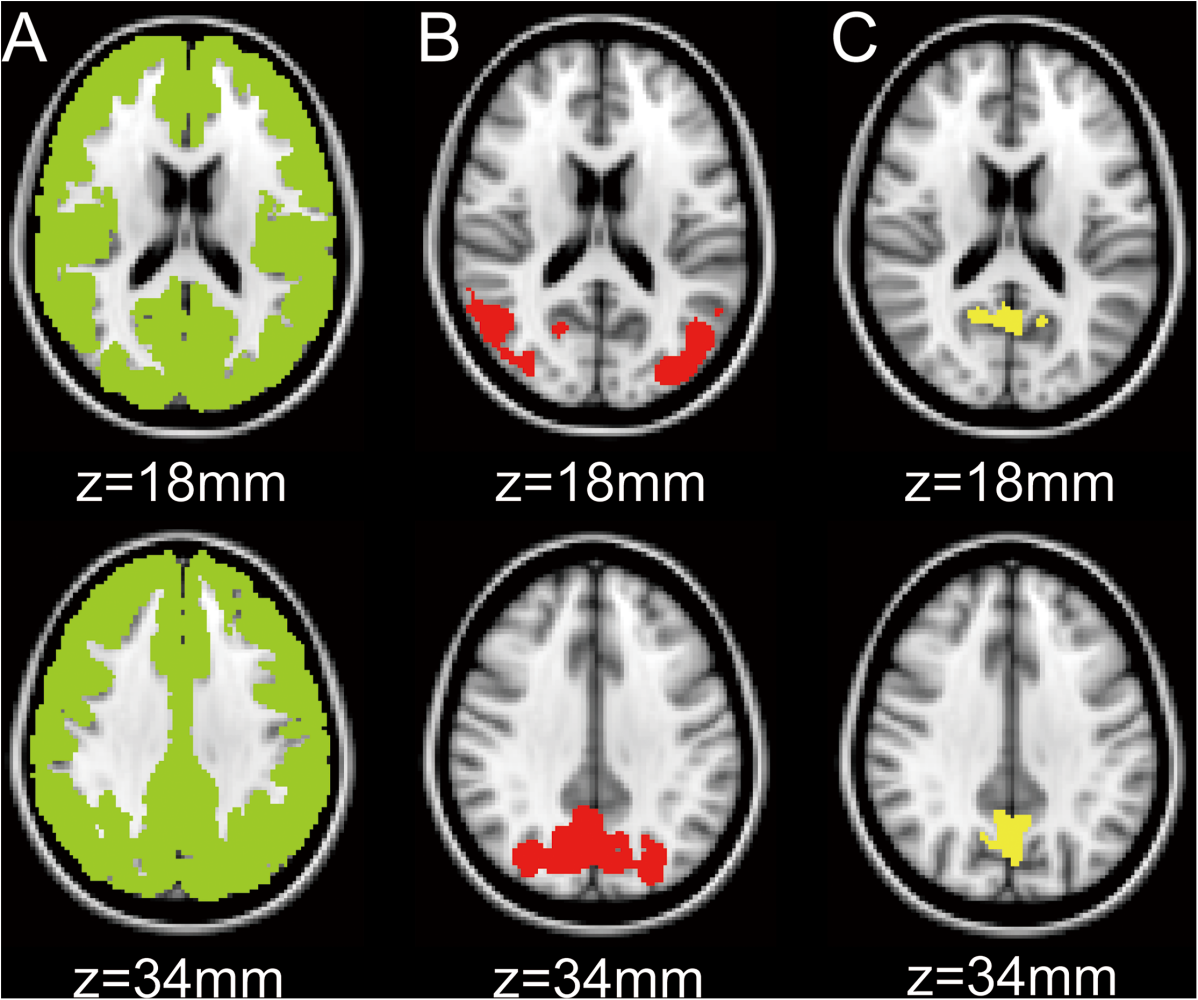
Volumes-of-interest (VOIs) in the MNI space. A mask of the whole cerebral cortex (A) and the two VOIs (B and C) are displayed on the axial sections of the MNI standard brain. The VOI for B was equal to the significant cluster detected by the voxelwise analysis. The VOI for C was placed on the precuneus, which was sampled from the Harvard-Oxford atlas. MNI: Montreal Neurological Institute.

Images were processed using the FMRIB Software Library version 5.0.4 (FSL; Oxford University, Oxford, UK) and the Statistical Parametric Mapping, version 12 (SPM12; Wellcome Trust Center for Neuroscience, London, UK) implemented in MATLAB, version R2014a (The MathWorks, Natick, MA). Using the in-house developed ^18^F-FDG template, all ^18^F-FDG images were transformed into the Montreal Neurological Institute (MNI) space from native space for voxelwise and subsequent volume-of-interest (VOI) analyses. Then, the whole cerebral cortex in each ^18^F-FDG image was masked with the MNI structural atlas included in FSL (Fig 1A). Using the mask as a reference region, normalized ^18^F-FDG images were created in the MNI space, and normalized values of ^18^F-FDG uptake were expressed as FU. The mean FU value was set as one in the whole cerebral cortex.

### Data processing and statistical analysis

The relationships between the two variables from age, plasma glucose levels, plasma insulin levels, and HOMA-IR were tested by a partial correlation analysis with gender adjustment. Statistical significance was set at two-tailed *p* < 0.05.

After applying 8 mm FWHM spatial smoothing to FU images, whole-brain voxelwise regression analysis was performed to assess the relationships between FU values and each of the three variables (i.e., glucose, insulin, and HOMA-IR), controlling the effects of age and gender. The null hypothesis was that ^18^F-FDG uptake did not negatively correlate with plasma glucose levels, plasma insulin levels, or HOMA-IR. First, we calculated a statistical *t* map of negative contrast between FU values and each of the three variables, using age and gender as nuisance variables. To control a type I error, the correction for multiple comparisons was performed on the statistical map using the AFNI’s 3dClustSim program, which computes alpha levels denoting the probability of false positive clusters that are above the minimum cluster size for a specific voxelwise *p* value threshold (https://afni.nimh.nih.gov/pub/dist/doc/program_help/3dClustSim.html: accessed June 10, 2017). The corrected significance level was set at an alpha level of 0.05 with a voxelwise height threshold of *p* < 0.05.

Following the voxelwise analysis, two VOI analyses were performed in the MNI space. First, the significant cluster, which was detected by the voxelwise analysis, was used as a VOI (Fig 1B). Next, as a representative AD-related hypometabolic region, the precuneus VOI was sampled from the Harvard-Oxford atlas included in the FSL (Fig 1C). In each of the two VOI analyses, the relationships between FU values and each of the three variables (i.e., glucose, insulin, and HOMA-IR) were then tested by a partial correlation analysis with age and gender adjustment. The null hypothesis was that ^18^F-FDG uptake did not negatively correlate with plasma glucose levels, plasma insulin levels, or HOMA-IR values. All statistical analyses were conducted using SPSS Statistics version 22 (IBM, Armonk, NY). Statistical significance was set at one-tailed *p* < 0.05.

## Results

**Fig 2:**
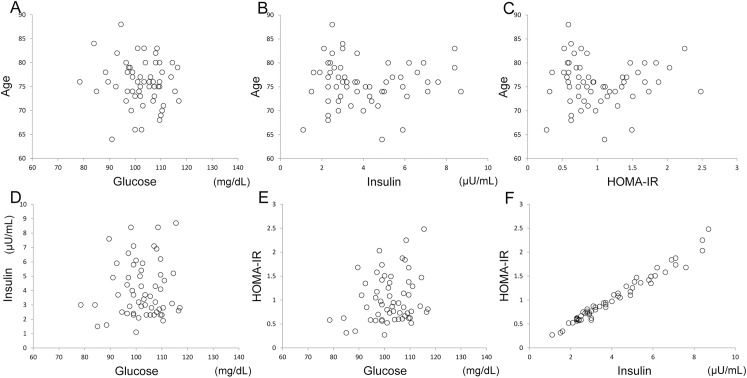
Sample age, glucose and insulin measurements, and insulin resistance. Relationships between age and glucose (A), between age and insulin (B), between age and HOMA-IR (C), between insulin and glucose (D), between HOMA-IR and glucose (E), and between HOMA-IR and insulin (F) are displayed. HOMA-IR: Homeostasis model assessment of Insulin Resistance.

Age, plasma glucose levels, plasma insulin levels, and HOMA-IR in all subjects were 75.7 ± 6.4 years, 102.2 ± 8.1 mg/dL, 4.1 ± 1.9 μU/mL, and 1.0 ± 0.5, respectively (mean ± standard deviation). The relationships among the four variables are shown in Fig 2. There was no significant correlation of age with glucose levels (partial correlation coefficient: *r* = -0.089, *p* = 0.51), insulin levels (*r* = 0.015, *p* = 0.91) or HOMA-IR (*r* = 0.016, *p* = 0.90). In addition, there was no significant correlation of glucose levels with insulin levels (*r* = 0.057, *p* = 0.67) or HOMA-IR (*r* = 0.209, *p* = 0.12). However, plasma insulin levels were significantly correlated with HOMA-IR (*r* = 0.985, *p* < 0.001).

**Fig 3:**
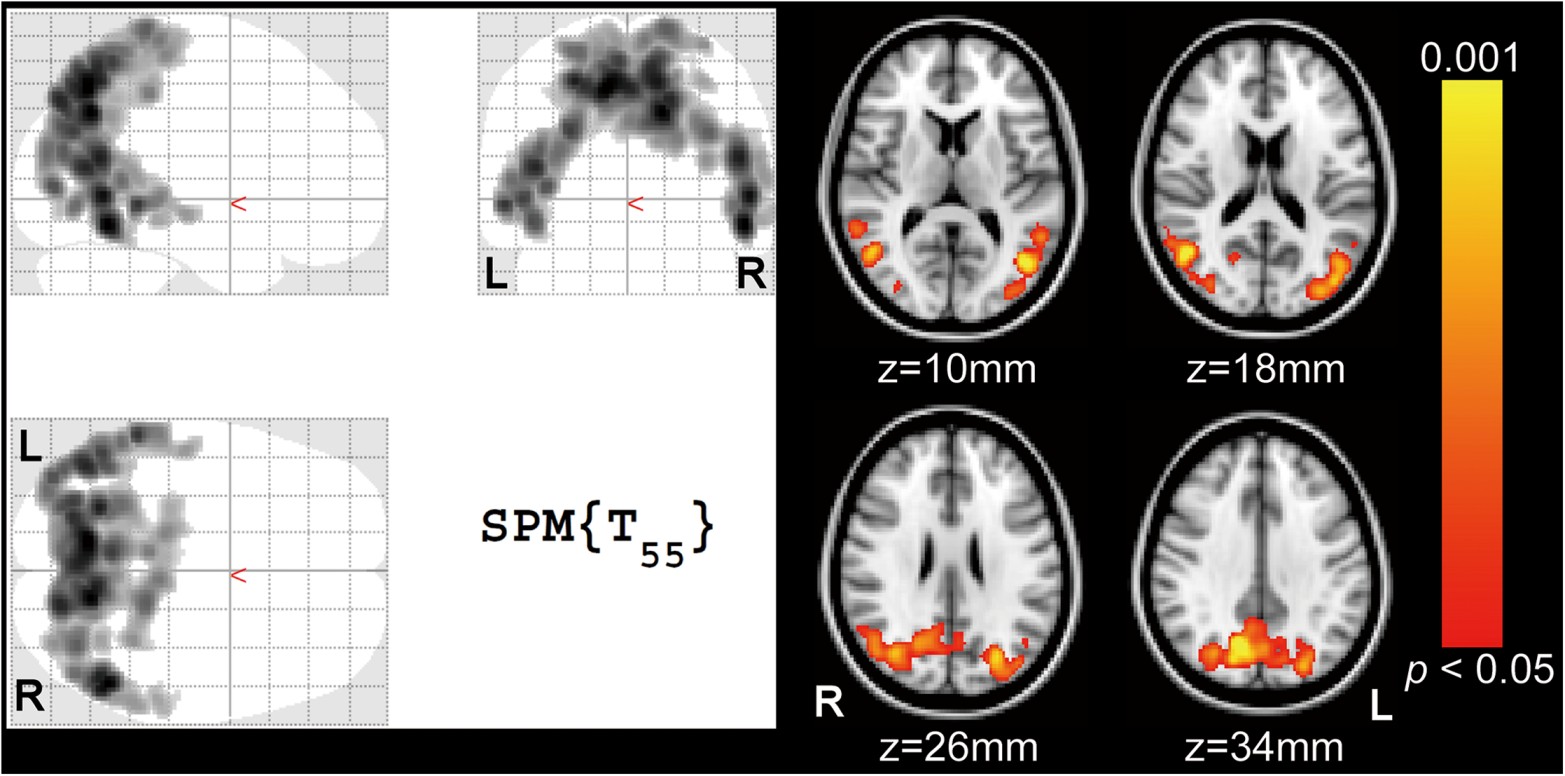
Negative correlation between plasma glucose levels and ^18^F-FDG uptake in whole-brain voxelwise analysis with age and gender adjustment. Significant clusters at a threshold of cluster-corrected *p* < 0.05 showing negative correlation of plasma glucose levels with FU values are displayed on the SPM glass brain (left side) and on the standard brain (right side) in the MNI space. They extended to the precuneus and lateral parietotemporal regions. The yellow–red scale represents the magnitude of *p* values. FU: normalized values of ^18^F-FDG uptake using the whole cerebral cortex as a reference region, R: right, L: left, SPM: Statistical Parametric Mapping, MNI: Montreal Neurological Institute.

Whole-brain voxelwise regression analysis with age and gender adjustments revealed a cluster consisting of 13220 voxels between plasma glucose levels and FU values (Fig 3) at a threshold of cluster-corrected *p* < 0.05, where ^18^F-FDG uptake decreased with increasing plasma glucose levels. They extended to the precuneus and lateral parietotemporal regions. However, voxelwise regression analysis revealed no significant correlation of FU values with plasma insulin levels or HOMA-IR at a threshold of cluster-corrected *p* < 0.05.

**Fig 4:**
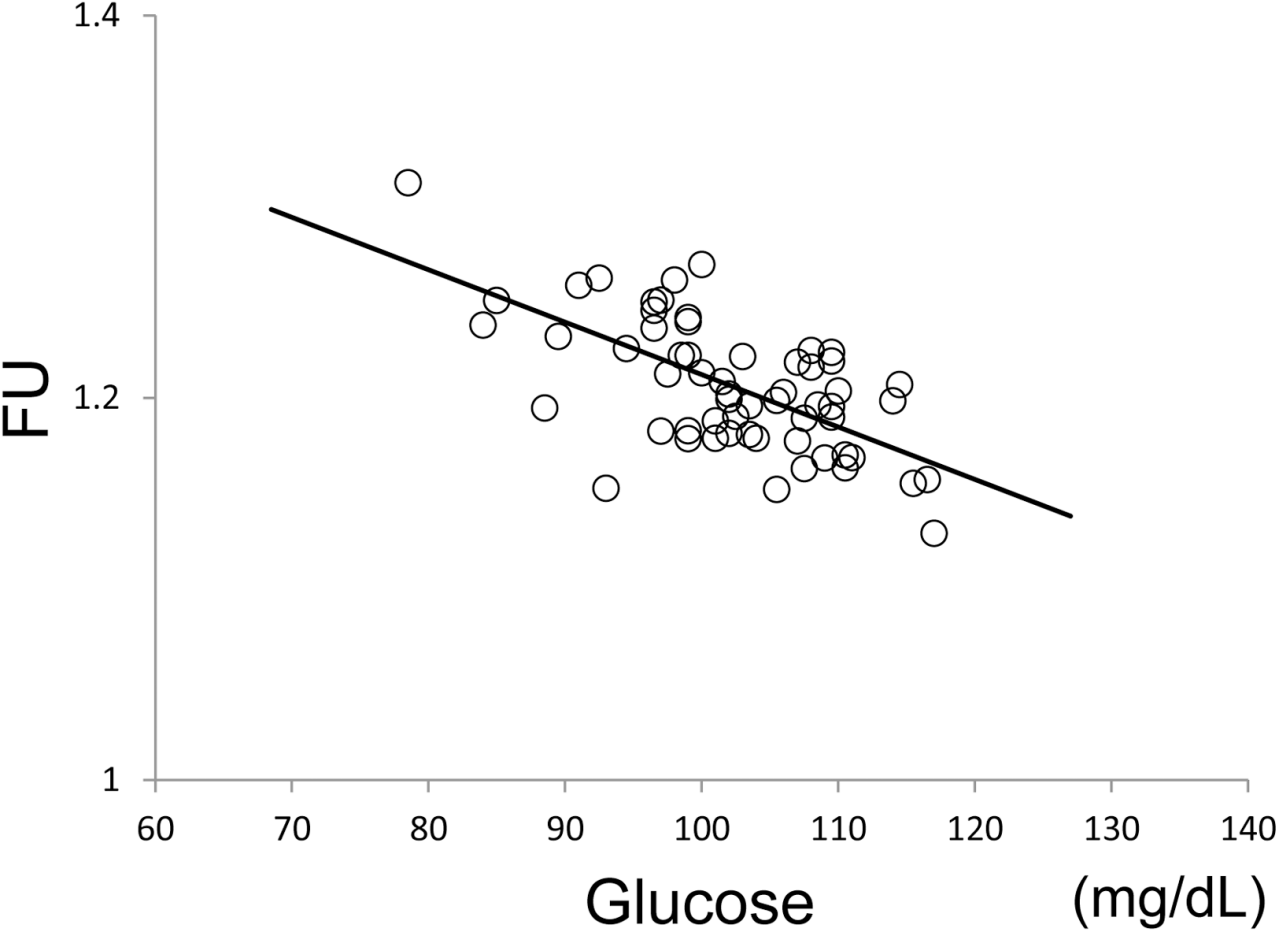
Relationship between plasma glucose levels and FU values in the significant cluster obtained from voxelwise analysis. The whole significant cluster shown in Fig 3 was used as a volume-of-interest (Fig 1B). A solid line represents the simple regression line between the two variables. FU: normalized values of ^18^F-FDG uptake using the whole cerebral cortex as a reference region.

**Fig 5:**
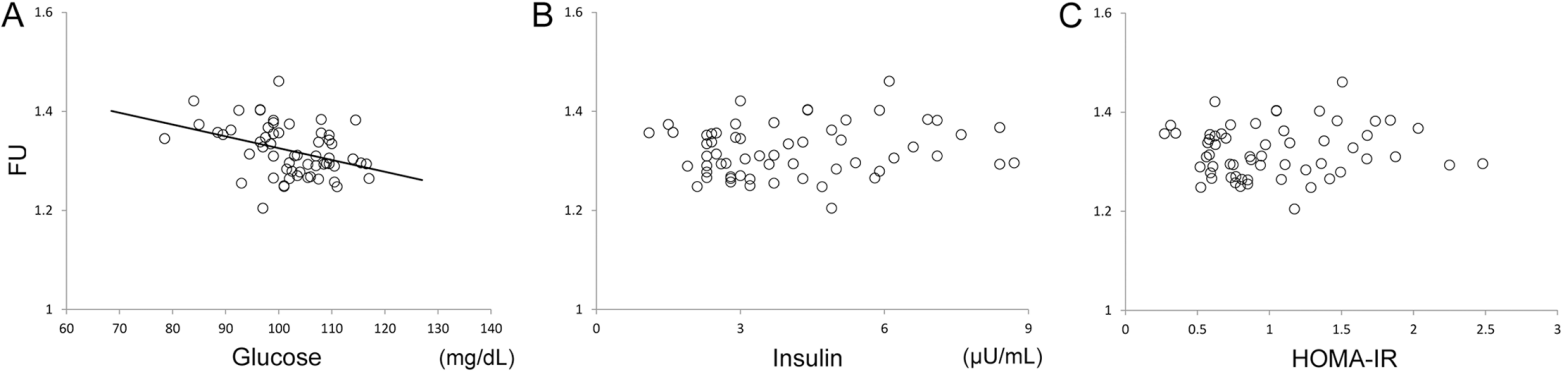
**Relationships of FU values with glucose (A), insulin (B), and insulin resistance (C) in the precuneus.** The precuneus mask was used as a volume-of-interest (Fig 1C). A solid line represents the simple regression line between FU values and glucose. HOMA-IR: Homeostasis model assessment of Insulin Resistance, FU: normalized values of ^18^F-FDG uptake using the whole cerebral cortex as a reference region.

The results from VOI analysis in the significant cluster which was detected by the voxelwise analysis (Fig 3) are shown in Fig 4. The whole significant cluster was used as a VOI (Fig 1B). Plasma glucose levels were significantly correlated with FU values (*r* = -0.652, *p* < 0.001). The simple regression line show that ^18^F-FDG uptake in the significant cluster decreases by approximately 4–5% when plasma glucose levels increase by 20 mg/dL. The results from VOI analysis in the precuneus area (Fig 1C) are shown in Fig 5. Plasma glucose levels were significantly correlated with FU values (*r* = -0.376, *p* = 0.002). However, there was no significant correlation of FU values with insulin levels (*r* = 0.156, *p* = 0.12) or HOMA-IR (*r* = 0.096, *p* = 0.24).

## Discussion

The primary objective of this study was to compare the effects of plasma glucose levels, plasma insulin levels and HOMA-IR on the cerebral ^18^F-FDG distribution, leading to the appearance of the AD-like pattern in cognitively normal healthy subjects. We showed that there was a negative correlation between plasma glucose levels and normalized values of ^18^F-FDG uptake in the precuneus and lateral parietotemporal regions, which are the main components of AD-related hypometabolic areas. Although we used the whole cerebral cortex as a reference region to create normalized ^18^F-FDG images, the results were comparable even with the use of the cerebellum or the global brain as a reference region. In this study, HOMA-IR was highly correlated with plasma insulin levels but not with plasma glucose levels, probably because the intersubject variability in plasma insulin levels was much larger than that in plasma glucose levels. When the coefficient of variance (CV), which is calculated as (standard deviation / mean), was used as an index of the intersubject variability, the CVs in plasma glucose levels, plasma insulin levels and HOMA-IR were 0.08, 0.47, and 0.48, respectively. Thus, of the three parameters, plasma insulin levels and HOMA-IR were related, but plasma glucose levels were uncorrelated with the other two parameters.

A few studies have investigated the relationship between insulin resistance and ^18^F-FDG uptake in the brain. In a PET study on 23 patients with prediabetes and early diabetes, Baker and colleagues showed negative correlations between HOMA-IR and ^18^F-FDG uptake in the precuneus, posterior cingulate, and lateral parietotemporal regions, which are also the representative AD-related hypometabolic areas [8]. However, they could not find any significant correlation between the two variables in six control subjects. The authors addressed that this difference between patient and control groups might come from the difference in the range of HOMA-IR; its range was approximately one to 12 in the patient group, while HOMA-IR was around 2.4 in the control group. Willette and colleagues reported that higher HOMA-IR predicted lower ^18^F-FDG uptake in AD-vulnerable regions for patients with AD but not for healthy subjects [9]. Another study from Willette and colleagues showed that higher HOMA-IR was associated with lower ^18^F-FDG uptake in the AD-related hypometabolic regions in cognitively normal late middle-aged adults at risk for AD whose range of HOMA-IR was 0.5 to 14.1 [10]. These findings suggest that the relationship between insulin resistance and ^18^F-FDG uptake in the brain can be different between healthy subjects and patients with diabetes or AD, and that our results from the healthy subjects with HOMA-IR range of 0.3 to 2.5 are not inconsistent with these previous studies. To our knowledge, this is the first study that has compared the effects of plasma glucose levels, plasma insulin levels, and HOMA-IR on the cerebral ^18^F-FDG distribution. Our study has provided evidence that, of the three parameters, plasma glucose levels strongly influence the cerebral ^18^F-FDG distribution in the precuneus and lateral parietotemporal regions, and has the greatest effect on the appearance of the AD-like pattern.

In general, as ^18^F-FDG competes with glucose for the glucose transporters and hexokinase [13], increasing plasma glucose levels absolutely reduces ^18^F-FDG uptake in every tissue of the global brain [14–17]. However, the magnitude of reduction in ^18^F-FDG uptake with increasing plasma glucose levels can be different across tissues [1], and the cerebral ^18^F-FDG distribution pattern can change depending on plasma glucose levels [7]. There are two possible explanations as to why increasing plasma glucose levels can alter the cerebral ^18^F-FDG distribution pattern. First, the alteration may be affected by the regional difference in the expression of glucose transporters and hexokinase and/or the regional difference in the magnitude of competition between glucose and ^18^F-FDG. Second, the alteration may be caused by the changes in regional neuronal activity because regional ^18^F-FDG uptake is associated with regional glucose metabolism [18]. We have conducted two studies to answer this issue. In one study, ^18^F-FDG and ^15^O-H_2_O PET scans were performed on nine young healthy volunteers in the fasting and glucose loading conditions [5]. ^15^O-H_2_O uptake reflects regional cerebral blood flow and regional neuronal activity, independently of glucose transporters and hexokinase. The study showed that glucose loading can alter the distribution pattern of ^18^F-FDG as well as ^15^O-H_2_O, and that uptake of both radioligands decrease in the AD-related hypometabolic regions. In the other study, dynamic ^18^F-FDG PET scans with arterial blood sampling, which directly measured regional glucose metabolism, were performed on 12 young healthy volunteers in the fasting and glucose loading conditions [6]. The study showed that glucose loading can decrease net glucose metabolism in the AD-related hypometabolic regions, although the magnitude of its reduction is very small. These studies strongly indicate that increasing plasma glucose levels can decrease regional neuronal activity in the AD-related hypometabolic regions.

The extension of the cluster in Fig 3 is similar to the functional anatomy of default mode network (DMN), which is characterized by high activity when the mind is not engaged in specific behavioral tasks, and low activity during focused attention on the external environment [19, 20]. The DMN is known to play an important role in regulating complex cognition and behavior [21–23]. Interestingly, recent studies using functional magnetic resonance imaging (fMRI) showed that the functional connectivity of DMN can decrease in patient with diabetes [24–26]. On the other hand, the functional anatomy of DMN considerably overlaps with the main components of AD-related hypometabolic regions in ^18^F-FDG images. Its functional connectivity is impaired in patients with AD [27], and its impairment becomes worse with AD disease progression [28]. These findings suggest that diabetes and AD share the vulnerability of DMN, and that reduced functional connectivity of DMN is associated with reduced neuronal activity in the AD-related hypometabolic regions. Therefore, these lines of evidence and our findings suggest that increasing plasma glucose levels may decrease the functional connectivity of DMN, leading to the reduced neuronal activity in the DMN-related components followed by the appearance of the AD-like pattern in ^18^F-FDG images. Further investigations with a combination of ^18^F-FDG PET and resting-state fMRI are required to assess this suggestion.

The precuneus and lateral parietotemporal regions shown in Fig 3 are similar to the areas identified as hypometabolic in patients with AD. However, it should be addressed that the magnitude of reduction in ^18^F-FDG uptake is quite different between the two situations. Glucose metabolism in patients with AD, whose MMSE scores were around 18, decreased by roughly 40%, compared to controls [29]. Another study showed that ^18^F-FDG uptake in patients with AD, whose MMSE scores were around 20, decreased by roughly 25%, compared to controls [4]. On the other hand, our findings showed that ^18^F-FDG uptake decreased by approximately 4–5% when plasma glucose levels increased by 20 mg/dL. Although it may be difficult to point out a 4–5% reduction in ^18^F-FDG images by visual inspection, statistical VOI or voxelwise analysis can find the difference. We therefore recommend that when statistical analysis is performed to assess ^18^F-FDG uptake, plasma glucose levels should be matched between groups or be set as a covariate to remove the effects of glucose on the cerebral ^18^F-FDG distribution.

The range of fasting plasma glucose in this study was 78.5 to 117 mg/dL. According to the criteria for diabetes, the levels of glucose < 100 mg/dL, 100 ≤ glucose < 126 mg/dL and glucose ≥ 126 mg/dL in the fasting condition are defined as normal, impaired fasting glucose (IFG), and diabetes, respectively, and individuals with IFG are defined as prediabetes [30]. Therefore, about half of the participants in this study might fall within the prediabetes range. Additionally, there is a possibility that some participants with higher glucose levels might be diagnosed with diabetes if we also measured values of the 2-hour oral glucose tolerance test and HbA1c. Interestingly, individuals with higher plasma glucose levels including IFG range are associated with cognitive decline measured by a battery of neuropsychological tests, and have a greater risk of developing cognitive impairment, compared to individual with non-diabetes [31–33]. Considering these known findings, some participants with higher plasma glucose levels might subclinically start to decrease cognitive function although all participants were deemed as non-MCI and non-dementia. Thus, this might affect the changes in the cerebral ^18^F-FDG distribution pattern in this study.

One of the limitations of this study was the lack of the MR-based partial volume correction, which might theoretically improve the accuracy of the results. However, the accuracy of MR-based partial volume correction is affected by several potential sources of error. These are misregistrations between the MRI and PET datasets, inaccurate estimation of resolution effects, missegmentation of the MRI data into brain tissue components, and potential heterogeneity of brain white matter [34]. Because of the low quality of MRI data, we could not apply the MR-based partial volume correction to the PET data. However, the method was not necessarily required in this study because of the following reasons. The participants were comprised of cognitively normal subjects who did not show the significant atrophy in the MRI findings. The intersubject variability in age of the participants was relatively small (CV = 0.06), and this study did not aim to compare the intergenerational differences. However, further investigations with the MR-based partial volume correction are desired to validate our findings.

## Conclusions

This study showed that plasma glucose levels have a negative correlation with ^18^F-FDG uptake in the precuneus and lateral parietotemporal regions, which are the main component of AD-related hypometabolic areas, and that ^18^F-FDG uptake decreased by approximately 4–5% when plasma glucose levels increased by 20 mg/dL. These findings confirmed that increasing plasma glucose levels can induce the appearance of the AD-like pattern in ^18^F-FDG images. However, there was no significant negative correlation of ^18^F-FDG uptake with plasma insulin levels or insulin resistance. Thus, of the three parameters, plasma glucose levels have the greatest effect on the appearance of the AD-like pattern in ^18^F-FDG images.
